# The role of different educational programs in specialty preference among Chinese medical students: a cross-sectional study

**DOI:** 10.1186/s12909-023-04701-7

**Published:** 2023-09-28

**Authors:** Shuangwen Wang, Xiaoqian Deng

**Affiliations:** 1 West China School of Medicine, Sichuan University, Chengdu, Sichuan China; 2 Department of Anesthesiology, Sichuan University West China Hospital, Chengdu, Sichuan China

**Keywords:** Educational Programs, Specialty, Medical students, Medical Education

## Abstract

**Background:**

The imbalanced supply of physicians in different specialties and the decreasing number of young doctors in China have made it important to understand specialty preference and influencing factors and to evaluate the role of different programs in specialty choice and career planning among Chinese medical students to help shape the social healthcare system and ensure adequate medical practitioners in each specialty.

**Method:**

A cross-sectional study comprising medical students from 5-year and 8-year program was conducted online. Demographics, specialty preferences and influencing factors, and career planning situations were collected and analysed by the chi-square test. Binary logistic regression analysis was performed in each program to identify the association between influencing factors and each specialty.

**Results:**

A total of 128 students (57.03% 5-year, 42.97% 8-year) responded to our survey. More 8-year students had a doctor in their household than 5-year students (25.45% vs. 10.96%, p < 0.05). The most preferred specialty among 5-year students was surgery, followed by others and internal medicine, while that most preferred by 8-year students was surgery, followed by internal medicine, obstetrics/gynecology and anesthesiology. Compared with 5-year students, more 8-year students considered ‘personal competencies’ (66.67% vs. 40.85%, p < 0.05), ‘the reputation of the specialty’ (18.52% vs. 7.04%, p ≤ 0.05), ‘fewer doctor‒patient disputes’ (27.78% vs. 11.27%, p < 0.05) and ‘advice from family members’ (24.07% vs. 7.04%, p < 0.05) influential. Among 5-year students, ‘personal competencies’ was positively associated with preference for surgery and ‘work-life balance’ was negatively associated. Among 8-year students, ‘personal competencies’ and ‘work-life balance’ were positively associated with internal medicine, while ‘interests in the specialty’ and ‘broad career prospects’ were negatively related. Many students need further career guidance, and personalized tutoring was the most wanted method.

**Conclusions:**

There was no difference between 5-year and 8-year students regarding specialty preference, but bias existed, possibly due to the influence of the real-world situation. Improving the working environment and welfare might be beneficial for developing a balanced distribution of the workforce among different medical departments. It is necessary for medical schools to develop programs accordingly to help them better plan their future careers.

## Background

Despite the fact that the doctor‒patient ratio in China has already met the WHO standard (2.3:1000) at 3.04:1000 [1, 2], there is still an imbalance in the supply of physicians in different specialties. In 2017, the number of psychiatrists per 100,000 residents was 2.19 in China, much lower than the number 13.06 in high-income country [3, 4]. The number of prehospital emergency medical personnel is still insufficient compared with China’s huge population base [5]. Additionally, the decline in medical students’ willingness to enter medical practice will make the situation even worse. The decreasing proportion of young doctors and the increasing proportion of doctors aged 60 years and older have also been witnessed in recent years [6]. Moreover, in a survey assessing the effect of the COVID-19 pandemic on medical students in China, 6.9% of students expressed a negative attitude towards entering medical practice in the future [7].

The choices made by medical students regarding their future are of prime importance for the development of the social healthcare system and ensuring adequate medical practitioners in each specialty [8], especially in times of undersupply of doctors [9]. Understanding the specialty preference of medical students and the influencing factors may shed light on developing policies and programs to attract students to pursue their dreams in those fields in short supply of professionals.

Bland et al. formulated a conceptual model of medical students’ choice of family medicine, which emphasized that in the process of career decision making, students needed to consider adaptation of social pressures, expectations of self and others, and perceptions of specialties [10]. Keisa et al. improved the model of career choice, and divided the influencing factors in to six dimensions: demographics and predisposition, financial and lifestyle considerations, health care environment, choice process and identity development, student interests relative to perceived specialty characteristics, and curriculum and school experience [11]. In conclusion, career choice was determined by students’ preferences and values, and influenced by their personal characteristics and educational program characteristics [12]. Other studies have evaluated the influencing factors students took into consideration when they decided their specialty. A systematic review and meta-analysis ranked the top 12 influencing factors [13]: individual-related factors, including academic interests and competencies, and contextual-related factors, including controllable lifestyles or flexible work schedules, patient service orientation, medical teachers or mentors, etc. Several surveys focused on medical students in China indicated that personal interest, clinical rotation experience, prospective specialty development, and future income were important factors influencing their decision-making [14]. Based on previous literature, associated factors were chosen according to the situation of Chinese medical undergraduates in the current study.

The educational program medical students study in, as an affecting factor in the model of career choice, might have an influence on the specialty choice among medical students. Generally, there is a 5-year medical bachelor’s degree program, a ‘5 + 3’ medical master’s degree program, and an 8-year medical doctoral degree program in China [15]. Medical programs in universities include the 5-year program and the 8-year program, making it possible to study the impact of different educational programs. For five-year students, the first two years cover the basics. The middle two years are spent studying clinical subjects, and the final year is dedicated to clinical rotations before they obtain a bachelor’s degree. If they want to further their study, they can also pursue a master’s or doctor’s degree after fierce competition in admission exams for another 3 or 6 years. For eight-year medical students, in later years of their studies, eight-year students would carry out some medical scientific research before they get a doctor’s degree. A survey published in Chinese found that the emphasis of future development was different between 5-year and 8-year students. While 5-year students persistently focused on graduate admission, 8-year students aimed at seeking desired tutors and job opportunities [16]. Additionally, more 8-year students planned to engage in a different career [16]. However, no studies have evaluated the role of different programs in specialty preference among medical students in China, thus the question remained unknown.

If students finally end up with a specialty they have no genuine interest in, it may harm the training of high-quality doctors [17]. However, a correct career choice will lead to higher satisfaction with work, better practice performance and improved healthcare standards [17]. What needs to be noted is that compared with the emphasis on medical knowledge and skills, current medical schools tend to show less interest in the education of career selection skills [18]. This highlights the need to understand the career planning situation of medical students and offer career guidance accordingly.

Therefore, the purposes of this study are to determine the specialty preference and influencing factors and evaluate the role of different programs in specialty choice and the career planning situation among Chinese students. It’s believed that current study might help policy makers and educators improve medical education to create a better medical environment and help medical students choose a more suitable career path.

## Methods

### Participants and data collection

A cross-sectional study was conducted to assess the specialty preference, factors influencing this choice and career planning among 5-year-program and 8-year-program undergraduate medical students in West China Medical School, Sichuan University. The questionnaire was published on the school website which every student could get access to in the period between August 2022 and September 2022. Students could respond to the questionnaire of their own free will. The study was approved by the ethics committee of West China Hospital and informed consent was obtained from all subjects participating in the study. Students were informed of the study goal and that participation was voluntary and anonymous. A total of 128 students responded to our survey.

### The survey

The questionnaire was designed by the study team and was sent for review by 3 medical students and 1 doctor to ensure the appropriate content and length before the final version was available online.

The survey was divided into three sections. The first section was about students’ demographics, including grade, gender, general performance, whether they are the only child, scholarship, whether they have published papers, whether they are pursuing a double bachelor’s degree or a bachelor’s degree with a minor, scholarship, whether they are student leaders, whether they are from urban or rural areas, monthly per capita income of household, whether there is a doctor in their household and parents’ highest educational level.

The second section inquired about students’ specialty preference and factors influencing their choice. They needed to choose one out of 10 specialties, which included surgery, internal medicine, obstetrics/gynecology (OB/GYN), pediatrics, ophthalmology, etc. The options ‘haven’t decided yet’ and ‘not gonna be a doctor’ were also available in this section. Then, for those who chose a certain specialty or ‘haven’t decided yet’, participants were asked to choose all factors associated with their desired specialty. Factors listed were interests in the specialty, personal competencies, broad career prospects, intellectual challenges, requirement for specific skill, anticipated income, less work pressure, work-life balance, the reputation of the specialty, not requiring much physical exertion, less interaction with patients, less doctor‒patient disputes, less competition, more job opportunities, advice from family members, advice from faculty members, the need of the society, personal life experience, and others.

The last section addressed career planning among students from 5-year and 8-year program. They were asked about their level of understanding of their preferred specialty when they made their choice, their level of understanding of themselves, their plans for the career, and whether they needed help to better plan their future.

### Statistical analysis

The Statistical Package for Social Sciences software (SPSS, version 26) was used to analyse the data. Differences in proportions were analysed using the chi-square test. Binary logistic regression analysis was performed to identify influencing factors associated with students’ willingness to choose a specialty as a career in 5-year and 8-year program. A P value less than 0.05 was considered statistically significant.

## Results

### Basic demographics

A total of 128 students responded to our survey (57.03% 5-year medical students, 42.97% 8-year medical students). The majority of respondents were fourth-year medical students (46.09%) in both programs (47.95% 5-year, 43.64% 8-year). No significant differences were identified between students from two programs in regards to general performance, scholarship, whether they have published papers, whether they are student leaders, monthly per capita income of household and parents’ highest educational level. However, there were 7 (9.59%) 5-year-program students who were pursuing a double bachelor’s degree or a bachelor’s degree with a minor, while none of the 8-year-program students did that (p < 0.05). More 8-year students had a doctor in their household than 5-year students (25.45% 8-year students vs. 10.96% 5-year students, P < 0.05). Table 1 demonstrates student demographics.

**Table 1:** Demographics of surveyed medical students

			5-year program (n = 73) n (%)	8-year program (n = 55) n (%)				P value*
**Grade**								
First			2 (2.74)	0				0.044
Second			4 (5.48)	2 (3.64)				
Third			11 (15.07)	20 (36.36)				
Fourth			35 (47.95)	24 (43.64)				
Fifth			21 (28.77)	9 (16.36)				
**Gender**								
Male			23 (31.51)	22 (40)				0.319
Female			50 (68.49)	33 (60)				
**General performance**								
Top 10%			17 (23.29)	10 (18.18)				0.398
Top 30%			18 (24.66)	14 (25.45)				
Top 50%			23 (31.51)	16 (29.09)				
Other			15 (20.54)	15 (27.28)				
**the only child**								
Yes			47 (64.38)	31 (56.36)				0.357
**scholarship?**								
Yes			48 (65.75)	42 (76.36)				0.193
**Have published papers**								
Yes			21 (28.77)	14 (25.45)				0.677
**Are pursuing a double bachelor’s degree or a bachelor’s degree with a minor**								
Yes			7 (9.59)	0				0.018
**student leader**								
Yes			41 (56.16)	36 (65.45)				0.288
**Where do you come from?**								
Big/medium city			16 (21.92)	21 (38.18)				0.097
Small city			49 (67.12)	27 (49.09)				
Rural areas			8 (10.96)	7 (12.73)				
**monthly per capita income of household (yuan)**								
Under 1000			5 (6.85)	3 (5.45)				0.801
1000–2000			6 (8.22)	3 (5.45)				
2000–5000			23 (31.51)	23 (41.82)				
5000–10,000			22 (30.14)	14 (25.45)				
10000-100,000			17 (23.29)	12 (21.82)				
Above 100,000			0	0				
**There is a doctor in my household**								
Yes			8 (10.96)	14 (25.45)				0.031
**Parents’ highest educational level**								
Primary school			5 (6.85)	1 (1.82)				0.531
Junior high school			2 (2.74)	4 (7.27)				
high school			14 (19.18)	9 (16.36)				
Junior college/bachelor’s degree			39 (53.42)	34 (61.82)				
Master’s degree			7 (9.59)	4 (7.27)				
Doctor’s degree			6 (8.22)	3 (5.45)				

*Chi-square test for the difference between the 5-year and 8-year program

### Specialty preference

In the survey, ‘surgery’ and ‘internal medicine’ comprised the 2 most common specialties chosen by medical students. A total of 14.84% of students were not decided regarding their career specialty. Additionally, 2.34% of students marked the option ‘not gonna be a doctor’ in the survey. Students showed the least interest in emergency medicine (0.78%). While 1.82% of 8-year students expressed interest in emergency medicine, no 5-year students did.

The most preferred specialty among 5-year students was surgery (32.88%), followed by others (12.33%) and internal medicine (9.59%), while that most preferred by 8-year students was surgery (25.45%), followed by internal medicine (20.00%), OB/GYN (9.09%) and anesthesiology (9.09%). The percentages of the choice ‘haven’t decided yet’ (12.33% vs. 14.84%) and ‘not gonna be a doctor’ (2.74% vs. 1.82%) were similar in 5-year and 8-year students. Specialty preferences among medical students by program are shown in Table 2.

**Table 2:** Specialty preference

	5-year program (n = 73) n (%)	8-year program (n = 55) n (%)	P value*
Surgery	24 (32.88)	14 (25.45)	0.238
Internal medicine	7 (9.759)	11 (20)	0.078
OB/GYN	5 (6.85)	5 (9.09)	0.441
Pediatrics	3 (4.11)	1 (1.82)	0.423
Ophthalmology	5 (6.85)	2 (3.64)	0.352
Emergency medicine	0	1 (1.82)	0.430
ENT	2 (2.74)	2 (3.64)	0.577
radiology/pathology/laboratory medicine	3 (4.11)	1 (1.82)	0.423
Anesthesiology	4 (5.48)	5 (9.09)	0.326
Other	9 (12.33)	2 (3.64)	0.075
Haven’t determined yet	9 (12.33)	10 (18.18)	0.250
Not gonna be a doctor	2 (2.74)	1 (1.82)	0.606
			0.492

*Chi-square test for the difference between the 5-year and 8-year program

### Factors that influence specialty choice

Removing students who chose not to be a doctor in the future, 98.18% of 8-year students and 97.26% of 5-year students completed the whole survey. Of the total 125 students (56.8% vs. 43.2%, 5-year vs. 8-year) who continued the survey, 77.6% of respondents rated ‘interests in the specialty’, 56% rated ‘broad career prospects’, 52% rated ‘personal competencies’ and 50.04% rated ‘anticipated income’ as influential on their preference of specialty (Table 3). Other less common factors influencing their career choice were ‘less competition’, ‘advice from faculty members’, ‘less interaction with patients’ and so on.

Generally, 8-year students will take more factors into consideration compared with 5-year students. Compared with 5-year students, more 8-year students considered ‘personal competencies’ (p < 0.05), ‘the reputation of the specialty’ (p ≤ 0.05), ‘fewer doctor‒patient disputes’ (p < 0.05) and ‘advice from family members’ (p < 0.05) influential. However, regarding those factors more often chosen by 5-year students, such as anticipated income and work-life balance, no significance was detected. Table 3 shows the factors influencing medical students from the two programs when they made their specialty choice.

**Table 3:** influencing factors

	5-year program (n = 71) n (%)	8-year program (n = 54) n (%)	P value*
Interests in the specialty	53 (74.65)	44 (81.48)	0.364
Personal competencies	29 (40.85)	36 (66.67)	0.004
Broad career prospects	38 (53.52)	32 (59.26)	0.522
Intellectual challenges	9 (12.68)	11 (20.37)	0.245
requirement for specific skill	16 (22.54)	12 (22.22)	0.967
Anticipated income	39 (54.93)	24 (44.44)	0.245
Less work pressure	17 (23.94)	16 (29.63)	0.475
Work-life balance	27 (38.03)	17 (31.48)	0.448
The reputation of the specialty	5 (7.04)	10 (18.52)	0.05
Not requiring much physical exertion	8 (11.27)	12 (22.22)	0.098
Less interaction with patients	7 (9.86)	6 (11.11)	0.082
Fewer doctor‒patient disputes	8 (11.27)	15 (27.78)	0.018
Less competition	3 (4.23)	3 (5.56)	0.730
More job opportunities	10 (14.08)	10 (18.52)	0.503
Advice from family members	5 (7.04)	13 (24.07)	0.007
Advice from faculty members	3 (4.23)	7 (12.96)	0.074
The need of the society	15 (21.13)	7 (12.96)	0.235
Personal life experience	20 (28.17)	9 (16.67)	0.131
Other	2 (2.82)	0	0.214

*Chi-square test for the difference between the 5-year and 8-year program

Table 4 shows the influencing factors and specialty preference after adjusting for other characteristics in students from the 5-year and 8-year program. For 5-year students, a high odds ratio (OR) for ‘personal competencies’ and a low OR for ‘work-life balance’ were associated with a preference for surgery. For 8-year students, ‘personal competencies’ and ‘work-life balance’ were positively associated with preference for internal medicine, while ‘interests in the specialty’ and ‘broad career prospects’ were negatively related.

**Table 4:** Influencing factors associated with preference among 5-year and 8-year students

	Odds ratio (95%CI)	P value		
5-year students				
Surgery				
Personal competencies	4.01 (1.04–15.50)	0.044		
Work-life balance	0.11 (0.01-1.00)	0.050		
8-year students				
Internal medicine				
Interests in the specialty	0.002 (0.000-0.125)		0.003	
Personal competencies	496.02 (2.954-83286.56)		0.018	
Broad career prospects	0.02 (0.001–0.438)		0.013	
Work-life balance	26.25 (1.462–470.93)		0.027	

Data were adjusted for all influencing factors in Table 3 by binary logistic regression analysis, and results with significance are presented

CI confidence interval

### Career planning situation

The survey showed that almost half of medical students (49.6%) only knew a little about the specialty at the time they chose it. Regarding the understanding of their interests, personalities and abilities, a majority of students (72.8%) thought that they knew themselves moderately rather than well. Although most students (85.19%) agreed that career planning is of great importance, only 12.8% of students had detailed plans and sticked to them, while a large number of students (67.2%) merely had vague plans for their careers. Many students (48.0%) thought that the career guidance courses in the college were just so-so and that they needed further guidance (58.4%). Personalized tutoring (41.6%) and specialty-related salons (35.2%) were the most welcoming methods to help shape their future. No significant difference was identified between 5-year and 8-year students among these variables. The data regarding their career planning are shown in Table 5.

**Table 5:** Career planning among medical students

	5-year program (n = 71) n (%)	8-year program (n = 54) n (%)			P value*
**How much did you know about the specific specialty when you decided to choose it?**					
Knowing well	8 (11.27)	3 (5.56)			0.374
Knowing some	28 (39.44)	18 (33.33)			
Knowing a little	33 (46.48)	29 (53.7)			
Not knowing	2 (2.82)	4 (7.41)			
**How much do you know your interests, personalities and abilities?**					
Knowing well	12 (16.9)	8 (14.81)			0.694
Knowing some	52 (73.24)	39 (72.22)			
Knowing a little	6 (8.45)	7 (12.96)			
Not Knowing	1 (1.41)	0			
**Do you think career planning is important?**					
Important	58 (81.69)	46 (85.19)			0.813
Kind of important	10 (14.08)	7 (12.96)			
Not important	2 (2.82)	1 (1.85)			
Have no idea	1 (1.41)	0			
**How’s your career planning situation?**					
I have a detailed plan and stick to it.	10 (14.08)	6 (11.11)			0.625
I have a detailed plan but haven’t put it into practice.	7 (9.86)	7 (12.96)			
I have a vague plan.	46 (64.79)	38 (70.37)			
I have no plan.	8 (11.27)	3 (5.56)			
**Is the career guidance courses in your college helpful?**					
Very helpful	5 (7.04)	6 (11.11)			0.483
Helpful	18 (25.35)	12 (22.22)			
Just so-so	37 (52.11)	23 (42.59)			
not very much	11 (15.49)	13 (24.07)			
**Do you need further help regarding career guidance?**					
Yes	41 (57.75)	32 (59.26)			0.749
No	14 (19.72)	8 (14.81)			
Whatever	16 (22.54)	14 (25.93)			
**What kind of help do you prefer?**					
Personalized tutoring	30 (42.25)	22 (40.74)			0.176
Specialty related salons	22 (30.99)	22 (40.74)			
Class	4 (5.63)	0			
Lectures	8 (11.27)	2 (3.7)			
Other	0	1 (1.85)			
I don’t need help.	7 (9.86)	7 (12.96)			

*Chi-square test for the difference between the 5-year and 8-year program

## Discussion

Understanding the specialty preference, influencing factors and the role of different programs are of great importance to help policy makers develop better education programs and regulations to improve the uneven and insufficient medical circumstance.

It is noted that the number of 8-year students who have a family member in medical practice is significantly higher than that of 5-year students, which might indicate that when these doctors’ younger generation feel like studying medicine, they tend to send these students to enter an 8-year program because most people think that 8-year students do not have to suffer the competition for the qualification of graduate admission and can obtain their doctor’s degree in a relatively short period of time. However, this program is not flawless, as a shorter training period might result in insufficient scientific and clinical practice [15], and 8-year students would have to expend extra effort to catch up to their peers from normal MD/PhD programs. For example, in a cross-sectional study focused on the research abilities of 8-year students, the majority of them felt pressured and had difficulties conducting research [19].

A previous study found that the most popular specialties among Chinese medical students were surgery and internal medicine, followed by obstetrics/gynecology and pediatrics [20]. Results of current study were slightly different, which suggested that surgery and internal medicine still ranked the top 2, but pediatrics was out of popular options. This was also contrary to surveys carried out in Uganda and Japan, which found that pediatrics ranked third of students’ choices [1, 21]. This declining may be due to the increasing incidents of medical violence and larger workload in pediatrics in recent years [22]. Taking the current situation of pediatrics into consideration, the declining willingness might worsen the insufficient supply of the workforce and highly educated practitioners and increase the work stress of pediatricians, leading to a vicious cycle.

Regarding influencing factors, the consideration of financial situation such as anticipated income, is a common concern for medical students to pursue their further specialized study, which might not only affect their choices of specialty, but even become an obstacle in their career path. For medical students in Togo, it might take up to 10 years for medical students to get financially ready to start their specialized training after general practitioner study [23].

Work-life balance has been taken into account by young doctors while choosing their specialized career [24]. For example, while many students were attracted to surgery and internal medicine for the financial rewards and attached prestige [24], other students strongly reject them due to the uncontrollable lifestyle [1, 25]. However, our study found that for 8-year students who took work-life balance into consideration, they were more likely to choose internal medicine as their future career. Being unable to maintain this balance might also be the reason why students were rejecting emergency medicine and pediatrics [5, 22]. The recruitment issues of these two specialties were also witnessed in UK [26, 27]. How to ensure a controllable lifestyle might be the key point to make such ‘shortage specialties’ more attractive to medical students.

Between 5-year and 8-year students, a significant difference was found regarding personal competencies, but was undetectable regarding anticipated income. This may indicate that students from both programs would take salary into consideration, but more 8-year students would evaluate the matching degree between the specialty and personal abilities compared with 5-year students. This result might offer a possible explanation for the previous study, which found that participants from the 8-year program gave the highest score regarding career and system satisfaction [15].

There are several ways to influence students’ preference towards certain specialties. First, developing specialized training to arouse students’ interest and sense of achievement is a viable method. After a 3-day intensive training on emergency medicine, both clinical knowledge and skills were enhanced, and some students showed their preference for seeking their future career in emergency medicine [28]. Moreover, designing special medical educational programs to cultivate specialized doctors could be possible. For example, a national medical educational program aiming to train physicians for rural areas was started in 2010, and well-trained students worked in township health centers as required [29, 30]. Additionally, as low salaries, poor working conditions, stress or large workloads, and burnout were some of the main reasons for attrition of health workers [31–34], improving the working environment and welfare of less welcome specialties might be helpful.

A thorough understanding of the characteristics of a certain specialty and personal needs is necessary to make a career decision [35]. Many news reports have shown that residents frequently terminated their residency programs or change their specialty, which might be the consequence of a lack of career selection skills [18]. A similar situation was detected in the current study that a majority of medical students did not thoroughly know about their chosen specialty when they made their decision and merely possessed a vague plan towards their future career. A study found that it’s important to start career planning education as early as possible [36]. Most students thought further guidance was needed, similar to a previous study in Korea [18]. For the expected guidance methods, most students chose personalized tutoring, probably for the convenience of consulting personal situations and avoiding peer counterparts [18]. Specialty-related salons ranked second, which indicated that students needed more information. Besides, it was found that informational support had positive influences on academic satisfaction among male nursing students, and might eventually encourage them to sustain their nursing profession [37].

Our study is the first to assess the role of different educational programs in specialty choice and influencing factors among Chinese medical students. However, it has several limitations. On the one hand, the questionnaire used in this study was not validated and based on previous literature and expert opinions, which might cause inherent biases. On the other hand, this study was conducted in a single center and may not be representative of the entire population of Chinese medical students. Future studies could assess the specialty choice and influencing factors in multiple centers in different regions of China to draw a more accurate picture of current medical students’ future careers.

## Conclusions

Overall, our study discovered that there was no difference between 5-year and 8-year students regarding specialty preference, but bias existed, possibly due to the influence of the real-world situation. Improving the working environment and welfare might be beneficial for developing a balanced distribution of the workforce among different medical departments. More 8-year students would take personal competencies into consideration than 5-year students, which might help them select a suitable specialty. The lack of career planning was discovered in both programs without a significant difference. It is necessary for medical schools to develop programs accordingly to help them better plan their future careers.

## Data Availability

The datasets used and analysed during the current study are available from the corresponding author on reasonable request.

## List of abbreviations

OB/GYN: Obstetrics/gynecology
OR: Odds ratio
